# Mediating effect of self-concealment between non-suicidal self-injury and internet addiction in college students: a cross-sectional study

**DOI:** 10.1186/s40359-023-01393-y

**Published:** 2023-11-21

**Authors:** Xushu Chen, Qin Ma, Xueping Peng, Haijun Yang, Zixiang Ye, Cui Yang, Changjiu He

**Affiliations:** 1 School of Nursing, Chengdu Medical College, Chengdu, China; 2 The Fourth People’s Hospital of Chengdu, Chengdu, China; 3 Zigong Fourth People’s Hospital, Zigong, China

**Keywords:** Internet addiction, Non-suicidal self-injury, Self-concealment, College students

## Abstract

**Background:**

Internet addiction, defined as uncontrolled behaviour resulting from the use of the Internet without the influence of addictive substances, which can seriously impair academic, occupational and social functioning. Non-suicidal self-injury, defined as self-injurious behaviour without the intent to die, and its addictive characteristics are similar to those of Internet addiction. Currently, there is a lack of research on the relationship between non-suicidal self-injury and Internet addiction. The purpose of this study was to examine the relationship between non-suicidal self-injury and internet addiction among college students and the role of self-concealment in this relationship.

**Methods:**

In this study, data were collected online between December 2022 and January 2023 from 600 university students in Chengdu, Sichuan Province, China, using purposive sampling. The questionnaires included the Non-Suicidal Self-Injury Inventory (NSSI), the Self-Concealment Scale (SCS) and the Internet Addiction Test (IAT).

**Results:**

A total of 573 valid questionnaires were recovered, with a valid recovery rate of 95.50%.

**Conclution:**

The results suggest that self-concealment plays a partial mediating role between non-suicidal self-injury and internet addiction among college students. The authors emphasized the importance of internet addiction. In order to reduce the occurrence of internet addiction, schools should provide targeted interventions to promote the psychological health of college students’ internet addictive behaviours.

## Introduction

With the continuous development of smart phones and high technology, Internet takes up most of students’ study and leisure time [1]. However, while the rapid development of the internet has brought convenience to students, it has also brought negative consequences that cannot be ignored, and the problem of internet addiction has attracted the attention of all sectors of society and has become a public health problem faced by young people around the world [2]. Internet addiction is a type of pathological or deviant internet use behaviour, which refers to a chronic or cyclical state of fascination caused by repeated use of the Internet by an individual, accompanied by psychological symptoms of addiction, such as increased tolerance and withdrawal reactions [3]. Students, as a group with a unique intuition and sensitivity to advanced technology and fairness, show a high level of enthusiasm and participation in the Internet. Due to weak external regulation and binding, it has become a common phenomenon for college students to spend a lot of time using smartphones [4]. Moreover, previous study found that 21.6% of college students develop internet addiction [5]. Another cross-sectional study conducted in China found that the detection rate of internet addiction among college students was 11.0% [6]. Internet addiction not only affects students’ academic performance, physical and mental health [7], and interpersonal interactions [8], but can also lead to serious harms such as delinquency [9]and suicide [10]. Improving internet addiction among students can help students return to normal study and life. Previous studies have shown that behavioural factors (non-suicidal self-injury) and (self-concealment) can strongly predict an individual’s internet addiction behaviour, but there is no study to confirm their combined effect on internet addiction. Therefore, in the context of the growing problem of internet addiction, it is necessary to explore the factors influencing internet addiction and their mechanisms of action among college students. As such, the purpose of this study is to examine the relationship between non-suicidal self-injury, self-concealment and internet addiction among college students, and to examine the mediating role of self-concealment in the relationship between non-suicidal self-injury and internet addiction among college students.

Non-suicidal self-injury refers to suicidal behaviour that deliberately injures or destroys body tissue without committing suicide. It includes direct injuries such as cutting, punching, burning, scratching bleeding areas and interfering with wound healing [11], and indirect injuries such as starvation, alcohol and drug abuse [12]. Although non-suicidal self-injury is not intended to kill, it can result in serious injury, unnecessary hospitalisation and even death [13]. Previous research has found that non-suicidal self-injury may be an important factor in internet addiction and may play a key role in promoting it [14]. Additonally, a growing body of evidence suggests that many adolescents who engage in self-injury go online, perhaps more than their peers who do not [15]. One study found that many young people who engage in non-suicidal self-injury go online, perhaps more than their peers who do not engage in non-suicidal self-injury. This suggests that there are more serious problems with internet use in this population. Students who engage in non-suicidal self-injury may have more psychological distress [14]. They may be more likely to relieve their distress through virtual networks [16]. This contributes to the development of internet addiction.

In addition, adolescents with non-suicidal self-injury may likely self-conceal due to the presence of shame and stigma [17, 18]. Self-concealment refers to the psychological tendency of individuals to actively conceal personal information from others that they perceive as painful or negative [13]. This information includes personal distressing experiences (non-suicidal self-injury), relationship problems, negative thoughts, etc. [19]. Self-concealment helps to maintain a positive self-image and avoid arousing resentment in others [20]. However, self-concealment also has a negative function, which is positively correlated with levels of depression, anxiety, and distress [21], and the greater the tendency to self-conceal, the greater the interpersonal distance [22]. In Lazarus’s coping model, self-concealment is viewed as an emotion-focused coping style that is avoidant in nature [23, 24]. This involves the intentional suppression of distressing information, thoughts, and feelings, which can prevent individuals from adopting a positive attitude. However, this avoidance of emotion-focused coping may hinder the use of problem-solving strategies. Self-concealment leads individuals to embrace internal coping strategies rather than external approaches [25]. High self-concealment students are less inclined to adopt problem-solving and help-seeking coping mechanisms [26], and more inclined to employ fantasy and patience when they face challenges such as be addicted to the Internet. Therefore, we hypothesised that self-concealment may mediate the relationship between non-suicidal self-injury and internet addiction.

Because of the anonymity and security of the internet, people with a high self-concealment tendency are more likely to engage in online behaviours to reveal secrets and relieve stress. This increases the risk of internet addiction [17, 27]. This is particularly true for those who see the internet as an escape from anxiety, depression or helplessness [28]. Non-suicidal self-injury behaviours can lead to stigmatisation of the individual. In order to minimise harm, individuals often engage in self-concealment. A high propensity for self-concealment then further increases the likelihood of developing internet addiction. Thus, students with non-suicidal self-injury behaviours may increase their internet addiction through increased self-concealment. Given the available evidence, we find it valuable to explore whether non-suicidal self-injury leads to internet addiction through the mediating effect of self-concealment, especially in the context of Chinese university students.

As such, and since no research is available on the matter to date [29]. Therefore, based on the literature review, we proposed the following four hypotheses after analysing the relationship between Internet addiction, non-suicidal self-injury and self-concealment in university students:

1. Non-suicidal self-injury has a direct positive effect on Internet addiction.
2. Non-suicidal self-injury has a direct positive effect on self-concealment.
3. Self-concealment has a direct positive effect on Internet addiction.
4. The relationship between Non-suicidal self-injury and Internet addiction is mediated by self-concealment.

Our study will help provide new research directions for mental health professionals to reduce internet addiction among students.

## Methods

### Study design

The cross-sectional study was conducted from December 2022 to January 2023 by collecting data from university students through the online questionnaire platform “Questionnaire Star”, and sampling university students from three universities in Chengdu City using the purposive sampling method. All students who completed the online survey were at least 18 years old and were considered to have consented to participate in this study by completing the online survey. To ensure an adequate sample size, we also referred to the sample size estimation formula for cross-sectional studies: $n = \frac{{{Z^2}P(1 - P)}}{{{d^2}}}$ [30]. where n is the sample size, *Z* is the statistic corresponding to the confidence level, *P* is the expected prevalence and d is the precision. We assume a confidence level of 95.0%. The detection rate of Internet addiction among 3738 college students in China was 19.2% with a precision of 5.0%, as reported by Wang et al. [31]. Considering the 20% questionnaire loss rate, the sample size was at least 375 cases. In the end, 600 questionnaires were distributed, and after manually eliminating 27 unreliable data, 573 valid questionnaires were finally obtained, and 95.5% (573/600) of valid responses were analysed.

### Qestionnaire

The questionnaire was divided into two main parts. The first part included the self-reported (gender, age, only child, student cadre, whether under study pressure, monthly living expenses, interpersonal relationships).The digital questionnaire was self-administered in Chinese, the native language of the participants, and took approximately 15 min to complete. The second part included the following scales.

### Measurement of non-suicidal self-injury

In this study, the Non-Suicidal Self-Injury (NSSI) scale developed by You et al [32] was used to assess whether participants had engaged in self-harming behaviours without suicidal ideation in the past 6 months and the frequency of these behaviours, which includes seven items such as self-cutting, burning, biting and so on. This questionnaire has using a 7-point Likert scale, 1= ‘never’, 2= ‘1–2 times’, 3= ‘once a month’, ‘4 = several times a month’, 5= ‘once a week’, 6= ‘several times a week’, 7= ‘almost every day’. The total score ranges from 7 to 49, with a total score above 7 indicating NSSI behaviour in this study. This scale has shown good reliability and validity in previous studies [33]. The Cronbach’s α coefficient of this scale in this study was 0.992.

### Measures of self-concealment

In this study, Larson et al. [21] developed the Self-Concealment Scale (SCS) to measure measure to assess the (a) self-reported tendency to keep things to oneself (e.g.,“There are lots of things about me that I keep to myself”); (b) possession of a personally distressing secret or negative thoughts about oneself that have been shared with few or no others (e.g.,“I have negative thoughts about myself that I never share with anyone”); and (c) apprehension about the disclosure of concealed personal information (e.g.,“If I shared all my secrets with my friends, they’d like me less”). Items on the SCS use a 5-point Likert scale, from strongly disagree to strongly agree. Possible scores range from 10 to 50, with higher scores reflecting a greater propensity toward self-concealment in this study. The Chinese version of the SCS is one-dimensional and this scale has good reliability and validity in previous studies [34]. The Cronbach’s alpha of this scale in this study was 0.921.

### Measurement of internet addiction

The Internet Addiction Test (IAT) developed by Young et al. [35] was used in this study. The scale consists of eight items such as (i) You feel preoccupied with using the Internet; (ii) You feel that you need to use the Internet for increasing amounts of time to achieve satisfaction; (iii) You have repeatedly made unsuccessful efforts to control, reduce, or stop using the Internet; (iv) You spend more time using the Internet than you originally intended; (v) You use the Internet as a way to escape from problems or to relieve a mood problem. each item has two options, ‘yes’ and ‘no’, and as long as at least five of the items are answered positively, it is considered to be internet addiction in this study. The scale has high reliability and validity in adolescents and Chinese high school students with a history of internet addiction [36, 37]. In this study, the Cronbach’s alpha coefficient of this scale was 0.805.

### Data collection procedure

With the consent of the school authorities, one researcher was selected from each school to receive uniform training. Students were invited to scan the Star QR code to access the questionnaire via mobile phone. Afterwards, the researchers explained unclear and ambiguous points raised by the participants during the fieldwork according to uniform guidelines. Note that all questionnaires are self-administered and completed separately by the participants.

### Statistical analysis

All analyses were performed using IBM SPSS statistical software version 25.0 (Armonk, NY, USA). The significance level of the two-tailed test was α = 0.05. Continuous data are presented as mean ± standard deviation and categorical data are presented as (n) and percentage (%). Pearson’s correlation analysis was used to test the correlation between variables. Similarly, McKinnon’s four-step method (46) was uesd to analysis the mediating role, which had to satisfy four specific criteria: (1) there was a significant correlation between the independent variable (NSSI) and the dependent variable (IA); (2) there was also a significant correlation between the independent variable (NSSI) and the mediator variable (self-concealment); and (3) after adjusting for the control of the independent variable (NSSI), the there is a significant correlation between the mediator variable (self-concealment) and the dependent variable (IA); (4) the indirect correlation coefficient between the independent variable (NSSI) and the dependent variable (IA) through the mediator variable (self-concealment) is significant. The first three steps were tested by linear regression equations with α_in_ = 0.05 and α_out_ = 0.01, respectively. Finally, the mediating effect was analysed using the PROCESS macro (Model 4) of SPSS version 3.3 [38]. Statistical significance was significanced when the 95% confidence interval did not contain 0.

## Results

### Common method biases test

To control for the problem of common method bias, this study used Harman’s one-way test for common method bias. The results showed that the first common factor explained only 24.91%(＜40%)of the variance. This indicates that there is no significant common method bias in this study despite the use of the questionnaire.

### Participant characteristics

Table 1 shows the socio-demographic characteristics of the students. Of the 573 eligible students, 416 (72.6%) were female and 157 (27.4%) were male. The mean age of the participants was (20.10 ± 1.46) years. 153 (26.7%) were only children and 420 (73.3%) had siblings. 185 (32.3%) were in the class council and 388 (67.7%) were not. 294 (51.3%) was under academic pressure. Of the students surveyed, the monthly living expense of most college students was between 1,000 and 2,000 RMB (78.5%). 129 participants (20.9%) had good interpersonal relationships, and the remaining 453 (79.1%) had difficulties with interpersonal relationships.

**Table 1:** Sociodemographic information of the participants. (n = 573)

Variable	Mean ± SD (range) N (%)
Gender	
Male	157(27.4)
Female	416(72.6)
Age	20.11 ± 1.44
Only child	
Yes	153(26.7)
No	420(73.3)
Student cadre	
Yes	185(32.3)
No	388(67.7)
Academic pressure	
Yes	294(51.3)
No	279(48.7)
Monthly living expenses	
＜1000 RMB	102(17.8)
1000～2000 RMB	450(78.5)
＞2000 RMB	21(3.7)
Interpersonal relationship	
good	120(20.9)
difficulty	453(79.1)

Correlation analysis of non-suicidal self-injury, self-concealment and internet addiction.

Table 2 shows Spearman’s correlations for the study variables. non-suicidal self-injury (r = 0.15, p < 0.01) and self-concealment (r = 0.25, p < 0.01) were significantly positively correlated with internet addiction. In addition, non-suicidal self-injury was significantly positively correlated with self-concealment (r = 0.20, p < 0.01). Thus, Hypothesis 1,2 and 3 were verified.

**Table 2:** Means, SDs, and correlations of all variables

Variables	Mean	SD	1	2	3
1. Non-suicidal self-injury	8.64	5.71	1		
2. Self-concealment	27.53	9.23	0.20**	1	
3. Internet addiction	3.70	2.33	0.15**	0.25**	1

** *p*＜0.01

The results of the hierarchical multiple regression.

**Fig. 1:**
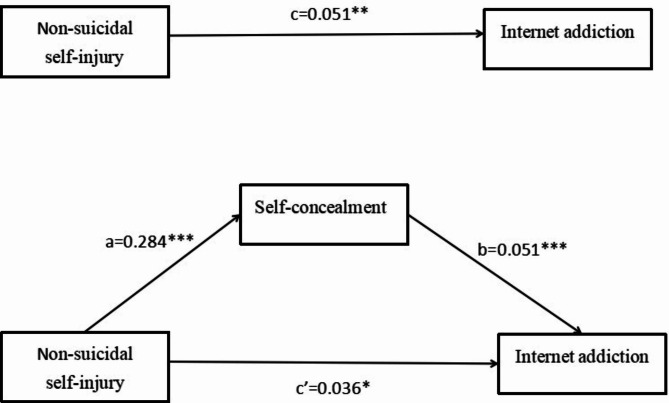
The hypothesised mediation model relating the effect of non-suicidal self-injury on Internet addiction through self-concealment (All coefficients are standardized coefficients)

Table 3 reports the analysis of mediating effects among the variables. After controlling for variables, there was a significant direct effect of non-suicidal self-injury on internet addiction (*β* = 0.051, *CI* [0.018, 0.084]). In addition, non-suicidal self-injury had a significant positive effect on self-concealment (β = 0.284, *CI* [0.153, 0.415]). self-concealment were significantly effected self-concealment (*β* = 0.051, *CI* [0.031, 0.072]). Furthermore, the effect of non-suicidal self-injury on internet addiction was statistically significant even when the mediating variable was included (*β* = 0.036, *CI* [0.003, 0.069]). Based on the bootstrap 95% *CI* not containing 0, it can be concluded that self-concealment partially mediates the relationship between non-suicidal self-injury and internet addiction. The mediation effect accounted for 29.41% of the total effect, confirming that self-concealment partially mediated the relationship between non-suicidal self-injury and internet addiction (Table 4), and Fig. 1 shows the final mediation model. Thus, Hypothesis 4 was proved.

**Table 3:** Regression analysis among study measures

Variables	*β*	*t*	*P*	LLCI	ULCI	*R* ^*2*^	*F*
**Result variable:** self-concealment							
Predictor non-suicidal self-injury	0.284	4.267	＜0.001	0.153	0.415	0.084	6.472
**Result variable:** internet addiction							
Predictor non-suicidal self-injury	0.036	2.150	0.032	0.003	0.069	0.092	7.453
Mediator self-concealment	0.051	4.873	＜0.001	0.031	0.072		
**Result variable:** internet addiction							
Independent variable non-suicidal self-injury	0.051	3.000	0.003	0.018	0.084	0.056	5.207

**Table 4:** Analysis of the mediating effect of self-concealment on non-suicidal self-injury and internet addiction

Paths	effect	S.E.	*P*	95%CI		effect ratio
				Low	High	
Total effect	0.051	0.017	0.003	0.175	0.841	100%
Direct effect	0.036	0.017	0.032	0.003	0.069	70.59%
Indirect effect	0.015	0.006	＜0.001	0.004	0.026	29.41%

95%CI for 95% confidence interval.

The hypothesised mediation model relating the effect of non-suicidal self-injury on Internet addiction through self-concealment(All coefficients are standardized coefficients).

## Discussion

This study used mediation to examine whether self-concealment mediates the relationship between non-suicidal self-injury and internet addiction among college students. It was found that non-suicidal self-injury among college students was positively related to internet addiction (see Hypothesis 1), and non-suicidal self-injury was positively related to self-concealment (see Hypothesis 2). Meanwhile, self-concealment was positively associated with internet addiction (see Hypothesis 3), and in addition, self-concealment may partially mediate (29.41%) the association between non-suicidal self-injury and internet addiction among college students in Chengdu (see Hypothesis 4). The findings of this study have theoretical and clinical implications.

### Non-suicidal self-injury and internet addiction

First, based on the results of this study, we demonstrate that non-suicidal self-injury had a significant effect on internet addiction among college students in Chengdu. This is line with previous findings [39]. Our findings may strengthen the link between non-suicidal self-injury and internet addiction. On the one hand, college students with non-suicidal self-injury behaviours may not be able to express or share their non-suicidal self-injury behaviours directly [34], and they may be more inclined to escape from the display and psychological distress by releasing it through the internet. On the other hand, due to the development and anonymity of the internet, most students with non-suicidal self-injury behaviours choose to express the purpose and nature of their non-suicidal self-injury through the internet [40].

### The mediating role of self-concealment

As hypothesised, our study shows that self-concealment mediates the relationship between non-suicidal self-injury and internet addiction, revealing the potential mechanism regarding how non-suicidal self-injury indirectly influences internet addiction. More importantly, non-suicidal self-injury not only has a direct effect on internet addiction, but also has an indirect effect through self-concealment. Indeed, the more serious and frequent students’ non-suicidal self-injurious behaviours are, the less they are willing to show their injuries and behaviours. On the one hand, since most students have high self-esteem, they care more about what others think and say about them [41]. At the same time, they are also worried that others will imitate their self-injurious behaviour if they know about it [42]. Therefore, students with non-suicidal self-injury behaviours are likely to self-conceal. On the other hand, lack of professional psychological help and social support may also increase the tendency of self-concealment [43, 44].

In the cognitive-behavioural model of pathological internet use, Davis [19] states that positive perceptions of the internet and negative perceptions of reality are key factors contributing to an individual’s internet addiction. In addition to these inappropriate perceptions, individuals with high levels of self-concealment may need more appropriate spaces to release psychological stress. Indeed, virtual cyberspace may give individuals a greater sense of security than face-to-face interpersonal interactions, making them more confident in disclosing information that is unfavourable to them. As a result, individuals with a high degree of self-concealment may feel safer and freer to expose themselves online in order to relieve the psychological pressure of prolonged secrecy. This is a negative reinforcement process that increases the likelihood of internet addiction. Moreover, students who engage in non-suicidal self-injury may be more likely to conceal both the self-harming behaviour and the cause of the self-injury, and a higher propensity for self-concealment may further encourage internet use, expression and disclosure, ultimately leading to internet addiction. These are possible explanations for the relationship between non-suicidal self-injury, self-concealment and internet addiction. In fact, our study reveals some mechanisms of action of non-suicidal self-injury on internet addiction, enriches the research on the relationship between non-suicidal self-injury and internet addiction and provides a new direction for internet addiction intervention for college students by reducing non-suicidal self-injury and self-concealment.

### Clinical implications

With the increasing prevalence of internet addiction among college students, it has become increasingly necessary to determine the root causes of these behaviours. Our findings revealed that non-suicidal self-injury was positively associated with internet addiction. Therefore, while focusing on internet addiction, attention needs to be paid to non-suicidal self-injury behaviours as well. While interventions to reduce addictive behaviours require a multi-layered approach, including determining individuals at risk. So individuals with high propensity for self-concealment need to be equally mitigated. In addition, it is recommended that psychologists begin by reducing self-concealment, which mediate the transition from non-suicidal self-injury to internet addiction. Finally, college students’ ability to cope with their problems and establish appropriate self-evaluations should be improved. At the same time, the state and government should provide professional psychological assistance and social support to reduce self-concealment. Additionally, active campus activities may help make more friends are conducive to reducing internet addiction.

## Conclusion

The study results suggest a need to address internet addiction among college students. It was discovered that students who engaged in non-suicidal self-injurious behaviours were more prone to self-masking, thereby increasing their likelihood of internet addiction. These findings highlight the importance for school educators to prevent and manage mental health issues, including internet addiction, among college students. Therefore, it is recommended that the state, government, and educational institutions establish a complete psychological intervention system. This system should provide professional treatment and support for the mental and behavioural health of university students, and reduce their dependence on the internet.

### Limitations

Our study has several limitations. Firstly, we tested only one mediating variable. The mediating effect was only 29.41% of the total effect. Therefore, caution must be exercised when promotion of research results. Future research needs to further explore the effects of other potential variables related to internet addiction, such as social support, stigma and self-efficacy. Secondly, this was a cross-sectional study and causal relationships between the study variables could not be inferred. Third, all study measures were self-reported and may be subjectively biased. Additionally, this study is quantitative in nature and it is necessary to conduct a qualitative study to ensure an in-depth understanding of the effects of internet addiction on students. Finally, the sample size of this study only included students from three universities in Chengdu. Due to the limited sample size, it is necessary to use a multi-centre and more representative sample to test and summarise the causal relationships between the variables obtained in this study.

## Data Availability

The raw data supporting the conclusions of this article will be made available by the authors, without undue reservation.

## Abbreviations

NSSI: Non-Suicidal Self-Injury scale
SCS: self-concealment scale
IAT: Internet addiction test
